# Disproportionate Decline in Trabecular Bone Score Compared to Bone Mineral Density in Southeast Asian Patients with Thalassemia: A Matched Control Study

**DOI:** 10.1007/s00223-026-01483-0

**Published:** 2026-02-09

**Authors:** Chatlert Pongchaiyakul, Nattiya Teawtrakul, Daris Theerakulpisut, Dueanchonnee Sribenjalak, Nipith Charoenngam

**Affiliations:** 1 Division of Endocrinology and Metabolism, Department of Medicine, Faculty of Medicine, Khon Kaen University, Khon Kaen, Thailand; 2 Division of Hematology, Department of Medicine, Faculty of Medicine, Khon Kaen University, Khon Kaen, Thailand; 3 Division of Nuclear Medicine, Department of Radiology, Faculty of Medicine, Khon Kaen University, Khon Kaen, Thailand; 4 Division of Endocrinology, Massachusetts General Hospital, Harvard Medical School, 50 Blossom St, Boston, MA 02114 USA; 5 Department of Medicine, Faculty of Medicine Siriraj Hospital, Mahidol University, Bangkok, Thailand

**Keywords:** Thalassemia, Bone quality, Bone mineral density, Trabecular bone score

## Abstract

We aimed to assess trabecular bone score (TBS) alongside lumbar spine (LS) and femoral neck (FN) bone mineral density (BMD) in patients with transfusion-dependent thalassemia (TDT) and non-transfusion-dependent thalassemia (NTDT) compared with healthy controls. A cross-sectional study was conducted involving adult thalassemia patients and healthy controls matched at a 2:1 ratio by age and sex. LS and FN BMD and TBS were measured using dual-energy X-ray absorptiometry. Univariate and multivariate analyses were performed to identify factors influencing TBS. The study protocol was approved by the institutional ethics committee. Eighty-six thalassemia patients (mean ± SD age 32.2 ± 11.9 years; 60.5% women) and 172 healthy controls (mean ± SD age 33.7 ± 10.9 years; 60.5% women) were included. Thalassemia patients had significantly lower BMI, LS Z-score, FN Z-score and TBS than controls (all *p* < 0.001). Only 24.4% of thalassemia patients had normal TBS (≥ 1.350) compared to 77.9% of controls, with a tenfold higher prevalence of degraded microarchitecture (TBS < 1.200). Although TDT patients exhibited more severe disease features than NTDT patients, TBS was similarly reduced in both groups. In multivariate analysis adjusted for age, sex, BMI, and LS BMD, TDT status remained independently associated with lower TBS (β = − 0.047; 95% CI: − 0.083 to − 0.012). No significant correlations were found between TBS and hemoglobin, serum ferritin, lactate dehydrogenase or glucose levels. This study provides new evidence that TBS was significantly impaired in Southeast Asian patients with thalassemia, independent of BMD, with a disproportionate reduction observed particularly in TDT patients.

**Supplementary Information:**

The online version contains supplementary material available at 10.1007/s00223-026-01483-0.

## Introduction

Thalassemia is the most common inherited hematologic disorder globally, affecting over 10 million individuals [1]. It is characterized by impaired synthesis of alpha or beta globin chains in red blood cells, resulting in imbalanced hemoglobin production and chronic anemia [2, 3]. The condition is most prevalent in regions historically endemic for malaria, including Southeast Asia, Africa and the Mediterranean [1]. Clinically, thalassemia presents as a spectrum ranging from asymptomatic carrier states to severe disease, which can manifest early in life with profound anemia, skeletal abnormalities, and endocrine complications; the most severe form, hydrops fetalis, is often fatal in utero [3, 4]. Classification is based on disease severity, using terms such as thalassemia minor, intermedia and major, or by transfusion requirement as transfusion-dependent thalassemia (TDT) and non-transfusion-dependent thalassemia (NTDT) [3, 4].

Decreased bone mineral density (BMD) and increased fracture risk are well-recognized complications in thalassemia [5–9]. Several pathophysiologic mechanisms contribute to thalassemia-associated bone disease, including elevated pro-osteoclastogenic cytokines, disruptions in the Wnt/β-catenin signaling pathway, marrow expansion and multiple endocrinopathies secondary to iron overload (e.g., hypogonadism, diabetes, and hypoparathyroidism). Additional contributing factors include impaired growth hormone-insulin-like growth factor-1 signaling, vitamin D deficiency and reduced physical activity [10–16].

The development of trabecular bone score (TBS), a texture analysis derived from lumbar spine (LS) DXA images using 2D projection variograms, has enabled the assessment of bone microarchitecture, an important determinant of skeletal fragility independent of BMD [17]. We previously showed in a cohort of thalassemia patients that abnormal TBS and BMD were associated with a 14-fold higher likelihood of vertebral fracture, compared with a 9.2-fold higher likelihood associated with abnormal TBS alone and a 4.4-fold higher likelihood associated with abnormal BMD alone, suggesting potential utility of combining TBS and BMD for fracture risk prediction in this patient population [18]. Although thalassemia has been shown to impair bone microarchitecture, clinical data on bone quality have primarily been derived from studies in the Mediterranean and South Asian populations [19–21], with limited evidence from other regions. Furthermore, the relative impact of thalassemia on BMD versus bone quality (TBS) has not been well characterized. This study aimed to determine whether TBS provides additional information on bone microarchitecture beyond conventional BMD measurements in thalassemia patients, compared with age- and sex-matched healthy controls.

## Methods

We conducted an age- and sex-matched cross-sectional study comparing patients with thalassemia to healthy controls, using data from Srinagarind Hospital, Faculty of Medicine, Khon Kaen University, Thailand.

### Participants

Patients with thalassemia aged ≥ 18 years were prospectively enrolled between January 2013 and January 2014. All participants underwent standardized clinical evaluations, including medical history and physical examination by a physician. Clinical and laboratory data were collected. Transfusion-dependent thalassemia (TDT) was defined as thalassemia requiring lifelong regular red blood cell transfusions for survival, whereas non–transfusion-dependent thalassemia (NTDT) was defined as thalassemia not requiring lifelong regular transfusions, although intermittent/occasional transfusions may be administered in defined clinical situations [22, 23]. In our cohort, we operationalized ‘regular transfusion’ as transfusions typically administered at intervals < 8 weeks.

The control group was derived from a previously published cohort by our group that established normative trabecular bone score values. This cohort included 1,372 generally healthy, community-dwelling adults who underwent DXA testing at Srinagarind Hospital. From this dataset, a subset of controls was selected and matched to thalassemia patients at a 2:1 ratio based on age and sex, using an optimal pair matching algorithm implemented via the MatchIt function in RStudio version 2024.9.0.375 (RStudio, PBC, Boston, MA). This method minimizes the total absolute distance in covariate values between matched pairs. All thalassemia patients were successfully matched to appropriate controls.

The study protocol was approved by the Khon Kaen University Human Research Ethics Committee (Reference No. HE581241 and HE611419) and conducted in accordance with the Declaration of Helsinki and Good Clinical Practice Guidelines.

### Bone Mineral Density and Trabecular Bone Score Measurements

Dual-energy X-ray absorptiometry scans of the LS and hip were performed using a narrow fan-beam densitometer (Lunar Prodigy, GE Healthcare, Madison, WI) by radiologic technologists certified by the Thai Osteoporosis Foundation (TOPF) national certification. Furthermore, given the specific analysis software utilized, all technicians and interpreting physicians underwent mandatory, specific training modules on the CLARIO analysis software interface and procedures, which covers the BioClinical imaging guidelines for AGIOS AG348-C-018 and Gilead GS-US-216-0128 DXA Training prior to data collection for this study. All DXA images underwent visual inspection by the analyzing physician to ensure accurate bone edge detection. In cases where soft tissue density (e.g., hepatosplenomegaly) interfered with automatic edge detection, manual adjustment of the region of interest was performed to faithfully outline the vertebral body. Vertebrae with unresolvable artifacts or indistinct borders were excluded from analysis to ensure the validity of both BMD and TBS measurements. The interobserver coefficient of variation (CV) for areal bone mineral density in healthy individuals at our center was 1.5% for the LS, 1.3% for the femoral neck (FN) and 1.5% for TBS calculated from repeated measurement of 30 subjects. The least significant change for aBMD at our institution was 0.029 g/cm² at both the LS and total proximal femur. TBS values were derived from LS DXA images using TBS iNsight software (Med-Imaps, Pessac, France). Individual TBS values were obtained for vertebrae L1 through L4, and the mean TBS across these levels was used for analysis. TBS values were categorized as normal (≥ 1.350), partially degraded (1.200–1.350), and degraded (≤ 1.200) based on established reference standards [24].

### Statistical Analysis

Continuous variables are presented as mean ± standard deviation (SD), and categorical variables as frequency counts with percentages. Group comparisons of continuous variables were performed using the Student *t*-test for normally distributed data or the Mann-Whitney *U* test for non-normally distributed data. Categorical variables were compared using the chi-square test or Fisher’s exact test, as appropriate. Correlation analyses were conducted using Pearson’s correlation for normally distributed variables and Spearman’s rank correlation for variables violating normality assumptions. Multiple linear regression analysis was conducted to identify independent predictors of trabecular bone score. Box plots were used to illustrate group differences in continuous variables, while scatter plots were generated to visualize pairwise relationships among LS Z-score, FN Z-score, and TBS. Locally estimated scatterplot smoothing curves with 95% confidence intervals were fitted for the control group to illustrate expected trends. All statistical analyses and data visualizations were performed using RStudio version 2024.9.0.375 (RStudio, PBC, Boston, MA).

## Results

A total of 86 thalassemia patients (57 TDT and 29 NTDT) and 172 age- and sex-matched healthy controls were included in the analysis. The mean ± SD age was 32.2 ± 11.9 years for thalassemia patients and 33.7 ± 10.9 years for controls, with 60.5% of participants in both groups being women.

As shown in Table 1, thalassemia patients had significantly lower body mass index (BMI), LS Z-score, FN Z-score, and TBS compared with controls (all *p* < 0.001). Additionally, 37.2% of thalassemia patients had LS Z-scores below − 2, and 15.1% had FN Z-scores below − 2, whereas none of the controls fell below this threshold (*p* < 0.001 for both). TBS analysis revealed that only 24.4% of thalassemia patients had normal microarchitecture (TBS ≥ 1.350) compared to 77.9% of controls. In contrast, the proportions of patients with partially degraded (TBS 1.200–<1.350) and degraded (TBS < 1.200) microarchitecture were significantly higher in the thalassemia group (50.0% and 25.6%, respectively) than in controls (19.8% and 2.3%, respectively) (all *p* < 0.001).

**Table 1:** Characteristics of thalassemia patients and healthy controls

Variable	Thalassemia patients (*N* = 86)	Control (*N* = 172)	*P*-value
Age (years)	32.23 ± 11.91	33.67 ± 10.89	0.134
Sex (% Women)	52 (60.5%)	104 (60.5%)	1.000
Body mass index (kg/m^2^)	19.17 ± 2.19	22.24 ± 3.63	< 0.001
Lumbar spine Z-score	− 1.86 ± 0.98	− 0.25 ± 0.93	< 0.001
% Low lumbar spine Z-score (<-2)	32 (37.2%)	0 (0.0%)	< 0.001
Femoral neck Z-score	− 1.15 ± 0.81	0.45 ± 0.87	< 0.001
% Low femoral neck Z-score (<-2)	13 (15.1%)	0 (0.0%)	< 0.001
Trabecular bone score (TBS)	1.27 ± 0.13	1.42 ± 0.10	< 0.001
TBS Category: Normal (≥ 1.350)	21 (24.4%)	134 (77.9%)	< 0.001
TBS Category: partially degraded (1.200 – <1.350)	43 (50.0%)	34 (19.8%)	< 0.001
TBS Category: Degraded (< 1.200)	22 (25.6%)	4 (2.3%)	< 0.001

Table 2 demonstrates clinical and laboratory features between TDT and NTDT patients. TDT patients had significantly lower pretransfusion hemoglobin (7.2 ± 0.9 vs. 7.5 ± 1.7 g/dL, *p* = 0.012), higher serum ferritin levels (2690.1 ± 2458.2 vs. 1651.0 ± 1634.4 ng/mL, *p* = 0.038), and a markedly higher frequency of splenectomy (61.4% vs. 13.8%, *p* < 0.001). There were no statistically significant differences between the groups in BMI, lactate dehydrogenase (LDH), fasting glucose, or fracture history. Regarding genotype, most TDT patients had β-thalassemia/hemoglobin E (91.2%), while NTDT patients exhibited greater phenotypic heterogeneity, including β-thalassemia/hemoglobin E (51.7%), HbH disease (17.2%), HbH disease with HbCS (17.2%), and EABart’s disease (13.9%) (*p* < 0.001).

**Table 2:** Characteristics of thalassemia patients

Variable	TDT (*n* = 57)	NTDT (*n* = 29)	*P*-value
Age	30.6 ± 10.4	35.5 ± 14.1	0.217
Female (%)	38 (66.7%)	14 (48.3%)	0.157
Body mass index (kg/m^2^)	19.0 ± 2.2	19.5 ± 2.3	0.286
Pretransfused Hb (g/dL)	7.2 ± 0.9	7.5 ± 1.7	0.012
Serum ferritin (ng/mL)	2690.1 ± 2458.2	1651.0 ± 1634.4	0.038
Fasting blood glucose (mg/dL)	96.3 ± 56.0	89.9 ± 28.1	0.393
Lactate dehydrogenase (IU/L)	261.3 ± 138.6	305.3 ± 151.3	0.133
Splenectomy (%)	35 (61.4%)	4 (13.8%)	< 0.001
Fracture history (%)	23 (40.4%)	7 (24.1%)	0.211
Smoking (%)	6 (10.5%)	6 (20.7%)	0.339
Alcohol use (%)	8 (14%)	5 (17.2%)	0.941
Menopause (%)	11 (19.3%)	4 (13.8%)	0.765
*Phenotype group (%)*			
β-thal/Hb E	52 (91.2%)	15 (51.7%)	< 0.001
Homozygous β-thal	3 (5.3%)	0 (0.0%)	
HbH disease	0 (0.0%)	5 (17.2%)	
HbH disease with HbCS	0 (0.0%)	5 (17.2%)	
EABart’s disease^*^	2 (3.5%)	4 (13.9%)	

^*^Compound heterozygous Hb H and heterozygous Hb E

*P*-values represent comparisons between TDT and NTDT groups

TDT: transfusion-dependent thalassemia; NTDT: non–transfusion-dependent thalassemia; Hb: hemoglobin;β-thal/HbE: β-thalassemia/hemoglobin E disease; HbH: hemoglobin H; HbCS: hemoglobin constant spring

**Fig. 1:**
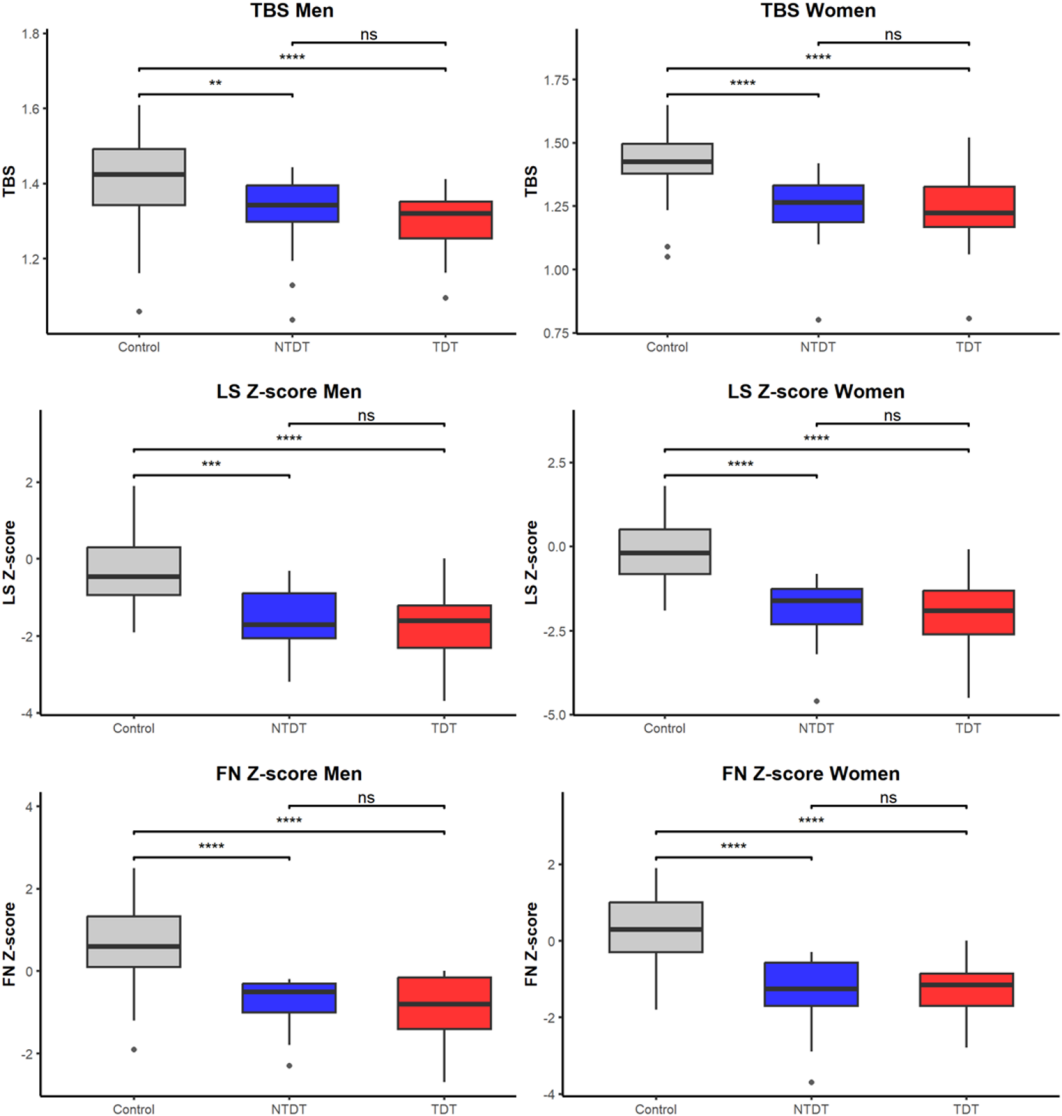
Box plot comparisons of trabecular bone score, lumbar spine Z-score and femoral neck Z-score among groups. Abbreviations: TBS: trabecular bone score; LS Z-score: lumbar spine Z-score; FN Z-score: femoral neck Z-score; TDT: transfusion-dependent thalassemia; NTDT: non–transfusion-dependent thalassemia.Statistical significance: *p* < 0.01 (**); *p* < 0.001 (***); *p* < 0.0001 (****)

Figure 1 and Supplementary Table show comparisons of trabecular bone score (TBS), lumbar spine (LS) Z-score, and femoral neck (FN) Z-score among controls, NTDT, and TDT patients, stratified by sex. In both men and women, controls had significantly higher TBS, LS Z-score, and FN Z-score compared with both NTDT and TDT groups (all *p* < 0.01). No statistically significant differences were observed between TDT and NTDT groups for any of these bone parameters.

To identify independent predictors of TBS, multiple linear regression analyses were performed (Table 3). In Model 1, which adjusted for age, sex, and BMI, thalassemia status was independently associated with lower TBS (β = − 0.143, 95%CI: − 0.174 – − 0.111). In Model 2, where TDT and NTDT were included as separate predictors, both were independently associated with lower TBS in a dose-dependent manner (TDT: β = − 0.151, 95%CI: − 0.186 – − 0.115; NTDT: β = − 0.127, 95%CI: − 0.173 – − 0.082).

**Table 3:** Multiple regression analysis of factors influencing trabecular bone score

Variable	Model 1		Model 2		Model 3	
	β	95%CI	β	95%CI	β	95%CI
Intercept	1.401^*^	[1.304, 1.499]	1.403^*^	[1.305, 1.501]	1.464^*^	[1.383, 1.545]
Age (per 1 year increase)	−0.002^*^	[− 0.003, − 0.001]	− 0.002^*^	[− 0.003, − 0.001]	− 0.003^*^	[− 0.004, − 0.002]
Women (vs. Men)	− 0.015	[− 0.043, 0.013]	− 0.014	[− 0.042, 0.014]	− 0.012	[− 0.035, 0.011]
BMI (per 1 kg/m^2^ increase)	0.004	[− 0.000, 0.009]	0.004	[− 0.000, 0.009]	0.003	[− 0.000, 0.007]
Thalassemia (vs. Control group)	− 0.143^*^	[− 0.174, − 0.111]	–	–	–	–
NTDT (vs. Control group)	–	–	− 0.127^*^	[− 0.173, − 0.082]	− 0.039	[− 0.081, 0.003]
TDT (vs. Control group)	–	–	− 0.151^*^	[− 0.186, − 0.115]	− 0.047^*^	[− 0.083, − 0.012]
LS Z-score (per 1 increase)	–	–	–	–	0.061^*^	[0.049, 0.073]

Model descriptions: Model 1: age, sex, BMI and thalassemia vs. control; Model 2: age, sex, BMI, TDT vs. control and NTDT vs. control; Model 3: age, sex, BMI, TDT vs. control, NTDT vs. control and LS Z-score

*Statistically significant at *p* < 0.05

TBS: trabecular bone score; BMI: body mass index; LS Z-score: lumbar spine Z-score; FN Z-score: femoral neck Z-score; TDT: transfusion-dependent thalassemia; NTDT: non–transfusion-dependent thalassemia

**Fig. 2:**
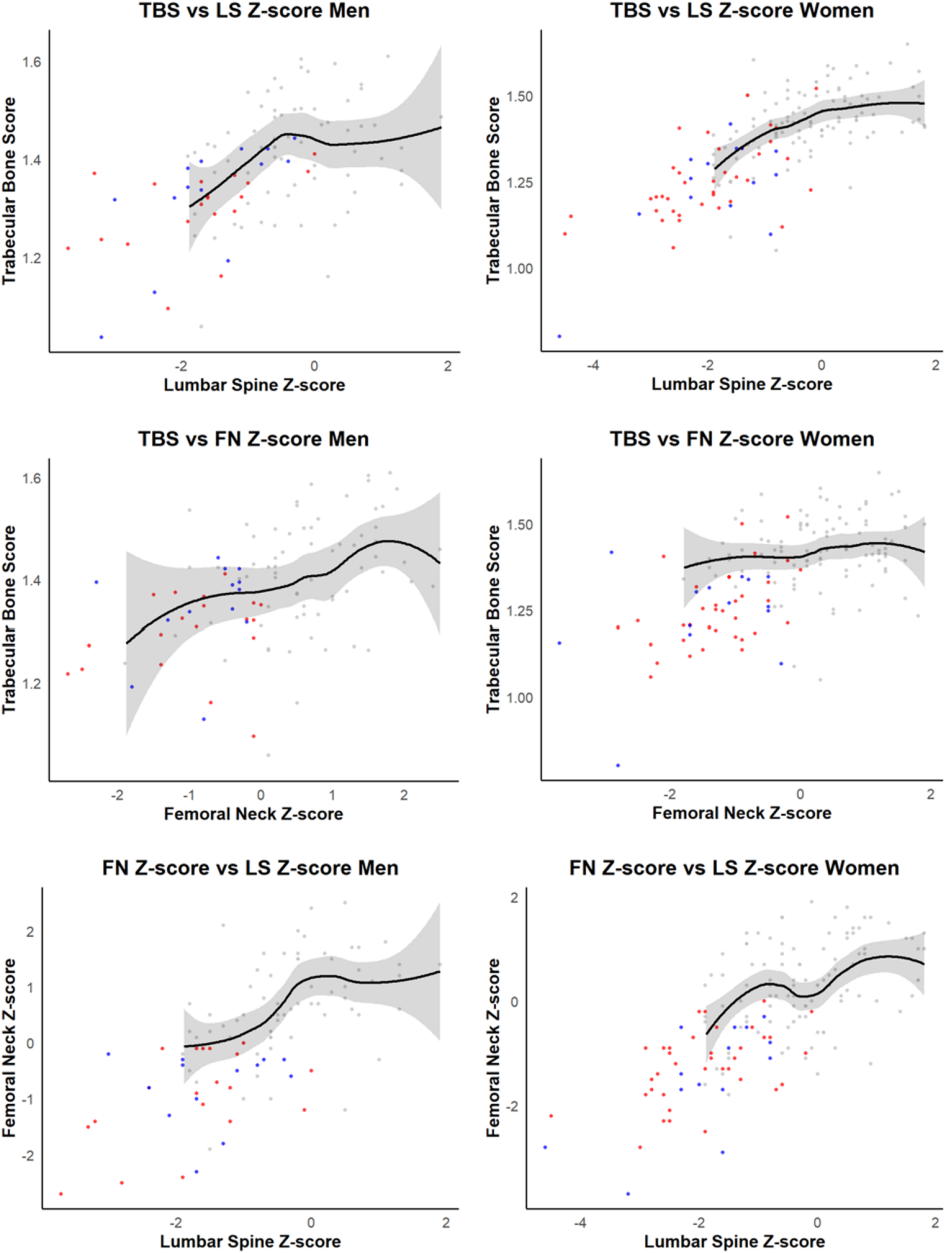
Pairwise relationships between trabecular bone score, lumbar spine Z-score and femoral neck Z-score in patients with thalassemia and controls curved lines and shaded areas represent locally estimated regression curves and their 95% confidence intervals for the control group. Red dots represent patients with transfusion-dependent thalassemia; blue dots represent patients with non–transfusion-dependent thalassemia; gray dots represent controls

To evaluate whether the reduction in TBS was disproportionately greater than expected based on BMD, Models 3, 4, and 5 additionally adjusted for LS Z-score, FN Z-score, and both LS and FN Z-scores, respectively. After adjustment for LS Z-score (Model 3), TDT remained significantly associated with lower TBS (β = − 0.047, 95% CI: − 0.083 − − 0.012), whereas the association with NTDT was attenuated and no longer statistically significant (β = − 0.039, 95% CI: − 0.081–0.003) (Table 3). When FN Z-score was included instead (Model 4), both TDT and NTDT remained significantly associated with lower TBS (TDT: β = − 0.080, 95% CI: − 0.120 – − 0.041; NTDT: β = − 0.052, 95% CI: − 0.101 – − 0.003). When both LS and FN Z-scores were included (Model 5), the associations of TDT and NTDT with lower TBS were attenuated and no longer statistically significant (TDT: β = − 0.033, 95% CI: − 0.071–0.004; NTDT: β = − 0.013, 95% CI: − 0.058–0.031; Supplementary Table 2). In an exploratory analysis stratified by transfusion dependence and splenectomy status (Supplementary Table 3), TDT patients with prior splenectomy (*N* = 34) had lower TBS and LS Z-score and a higher prevalence of fracture history compared with NTDT patients without splenectomy (*N* = 25) (TBS: 1.240 ± 0.120 vs. 1.292 ± 0.139; fracture history: 47.1% vs. 20.0%; LS Z-score: − 2.22 ± 1.02 vs. − 1.62 ± 0.88; all *p* < 0.05). Within TDT, splenectomized patients also had a lower LS Z-score than TDT patients without splenectomy (− 2.22 ± 1.02 vs. − 1.45 ± 0.79; *p* < 0.05). Markers of iron overload differed across groups: ferritin levels were higher in TDT patients with splenectomy than in NTDT patients without splenectomy (2860.76 ± 2744.57 vs. 1454.20 ± 1194.59; *p* < 0.05), and ferritin also differed between TDT patients without splenectomy and NTDT patients without splenectomy (2524.77 ± 1994.38 vs. 1454.20 ± 1194.59; *p* < 0.05). Interpretation of the NTDT/splenectomy subgroup (*n* = 4) is limited by small sample size. These findings suggest that trabecular microarchitectural deterioration in thalassemia, particularly in TDT, may be more pronounced than what is reflected by BMD alone. Figure 2 illustrates the pairwise relationships between TBS, LS Z-score, and FN Z-score across the three groups. Visual inspection suggests a non-linear pattern in the TBS-BMD relationships, with TBS appearing to decline disproportionately at lower BMD Z-scores, most notably among thalassemia women in the TBS versus FN Z-score plot. Correlation analysis between TBS and clinical variables in thalassemia patients showed no significant associations with hemoglobin (R^2^ = 0.02, *p* = 0.196), serum ferritin (R^2^ = 0.01, *p* = 0.393), fasting blood glucose (R^2^ = 0.01, *p* = 0.369), or lactate dehydrogenase (R^2^ = 0.01, *p* = 0.367).

## Discussion

This is the first study in a Southeast Asian population to evaluate TBS alongside LS and FN BMD in thalassemia patients compared with healthy controls. We found that Thai patients with thalassemia, including both TDT and NTDT, had significantly lower TBS, LS Z-scores, and FN Z-scores than age- and sex-matched controls. Notably, only 24.4% of thalassemia patients had a normal TBS, compared with 77.9% of controls, while the prevalence of degraded microarchitecture (TBS < 1.200) was more than tenfold higher in the thalassemia group. The mean TBS in TDT patients (1.24 in women and 1.30 in men) appears similar to the reported thresholds for fracture risk prediction and consideration of osteoporosis treatment in glucocorticoid-induced osteoporosis (approximately 1.24–1.31) [25]. These findings reinforce the high burden of skeletal fragility in thalassemia and highlight the importance of assessing bone quality in addition to BMD. Our findings extend prior work from the same thalassemia cohort, which examined predictors of vertebral fracture among thalassemia patients. In that report, low TBS and low BMD, particularly their combination, were strongly associated with vertebral fractures and endocrinopathies, and combined low TBS/low BMD remained independent predictors in multivariable analysis [18]. In contrast, the present study is novel in providing a matched case-control comparison and evaluating whether thalassemia status (TDT and NTDT) is associated with lower TBS beyond differences in BMD.

Although BMD is a well-established predictor of fracture risk in the general population [26], our results support growing evidence that BMD alone may not fully capture the extent of skeletal compromise in thalassemia. In our cohort, TBS was disproportionately reduced, particularly among TDT patients. Even after adjusting for LS or FN Z-scores in multivariate analysis (Models 3 and 4), TDT status remained independently associated with lower TBS, suggesting that trabecular deterioration exceeds what is expected based on BMD alone. This dissociation underscores the clinical utility of TBS in evaluating bone fragility and may help explain the high fracture rates in thalassemia.

These findings are consistent with previous studies from Mediterranean and South Asian cohorts reporting impaired bone quality in thalassemia [19–21]. In a study of 124 Italian patients with thalassemia major, TBS was significantly lower compared to 65 non-thalassemic controls undergoing DXA for evaluation of bone disease (mean ± SD: 1.04 ± 0.12 vs. 1.34 ± 0.11, *p* < 0.001), with further reductions observed in older and osteoporotic patients [19]. Similarly, in thalassemia intermedia, TBS was lower than in controls (1.22 vs. 1.36, *p* < 0.01), especially in those who were splenectomized, transfused or on iron chelation therapy [20]. Das et al. also demonstrated significantly reduced TBS (1.261 ± 0.072 vs. 1.389 ± 0.058) and microarchitectural deterioration on HR-pQCT in South Asian adults with TDT compared matched controls, including lower trabecular number, reduced cortical thickness and wider trabecular spacing [21].

Our study adds to the existing literature by being among the first to assess TBS in Thai thalassemia patients using a rigorously age- and sex-matched healthy control group and by including both TDT and NTDT subgroups. Notably, although TDT patients exhibited more severe clinical features, including higher ferritin levels and a greater prevalence of splenectomy (Table 2), TBS values were similarly impaired in NTDT patients (Fig. 1). The substantially lower BMI in thalassemia patients compared with controls may partly contribute to the observed TBS differences; although we adjusted for BMI in multivariable models, residual confounding related to body size and soft-tissue composition may persist and should be considered when interpreting these findings. Moreover, no statistically significant correlations were found between TBS and hemoglobin, serum ferritin, LDH, or glucose levels. While these null associations may reflect limited statistical power due to the small sample size, they may also underscore the multifactorial nature of thalassemia-associated bone disease. Mechanisms such as chronic anemia, marrow expansion, inflammatory cytokine activation and endocrine dysfunction likely contribute to skeletal impairment independent of transfusion intensity or iron overload [10–16]. Future studies incorporating comprehensive phenotypic characterization, including hormonal profiles, inflammatory markers, dietary history and physical activity, are warranted to better delineate the determinants of bone quality in this population.

The strengths of this study include the use of a well-characterized patient cohort, robust matching with a healthy control group, and the simultaneous evaluation of both BMD and TBS using standardized DXA-based techniques. However, certain limitations should be acknowledged. The relatively small sample size, especially within the NTDT subgroup, may have jeopardized our ability to detect modest associations and subgroup differences. Additionally, we did not collect systematic data on key bone-modifying factors such as endocrine status, physical activity, nutritional intake (including calcium and vitamin D) or inflammatory cytokines, all of which may mediate the association between thalassemia and impaired bone microarchitecture. Fracture outcomes were not systematically available in the matched control group; therefore, we could not perform a comparative assessment of fracture prediction using TBS and BMD in thalassemia versus controls, and fracture prediction was evaluated only in our prior report of the thalassemia cohort [18]. Lastly, as a cross-sectional analysis, our study cannot evaluate longitudinal changes in BMD and TBS over time.

## Conclusions

This study provides new evidence that trabecular bone score is significantly impaired in Southeast Asian patients with thalassemia, independent of BMD, with a disproportionately greater reduction observed particularly in TDT patients. These findings highlight the importance of incorporating TBS into bone health assessments for thalassemia and support the development of targeted strategies aimed at preserving both bone density and microarchitecture to reduce fracture risk in this high-risk population.

## Supplementary Information

Below is the link to the electronic supplementary material.

Supplementary Material 1
