# Supplementary material for: Disproportionate Decline in Trabecular Bone Score Compared to Bone Mineral Density in Southeast Asian Patients with Thalassemia: A Matched Control Study
Source: Calcif Tissue Int. 2026 Feb 9;117(1):24. doi: 10.1007/s00223-026-01483-0 (PMC12886205; doi:10.1007/s00223-026-01483-0)
Supplement: Supplementary file 1 — Supplementary Material 1 [file 223_2026_1483_MOESM1_ESM.docx]

**Supplementary Table 1. Comparison of trabecular bone score, lumbar spine Z-score and femoral neck Z-score among groups**

| Variable | Control  (N = 172) | TDT  (N = 57) | NTDT  (N = 29) | P-value | Comparison |
| --- | --- | --- | --- | --- | --- |
| TBS–All | 1.42 ± 0.10 | 1.26 ± 0.12 | 1.28 ± 0.14 | <0.001 | Control vs TDT |
|  |  |  |  | <0.001 | Control vs NTDT |
|  |  |  |  | 0.255 | TDT vs NTDT |
| TBS–Men | 1.41 ± 0.11 | 1.30 ± 0.08 | 1.32 ± 0.12 | <0.001 | Control vs TDT |
|  |  |  |  | 0.006 | Control vs NTDT |
|  |  |  |  | 0.193 | TDT vs NTDT |
| TBS–Women | 1.42 ± 0.10 | 1.24 ± 0.13 | 1.23 ± 0.15 | <0.001 | Control vs TDT |
|  |  |  |  | <0.001 | Control vs NTDT |
|  |  |  |  | 0.734 | TDT vs NTDT |
| LS Z-score–All | -0.25 ± 0.93 | -1.92 ± 0.99 | -1.75 ± 0.96 | <0.001 | Control vs TDT |
|  |  |  |  | <0.001 | Control vs NTDT |
|  |  |  |  | 0.464 | TDT vs NTDT |
| LS Z-score–Men | -0.34 ± 0.89 | -1.77 ± 0.99 | -1.61 ± 0.90 | <0.001 | Control vs TDT |
|  |  |  |  | 0.001 | Control vs NTDT |
|  |  |  |  | 0.63 | TDT vs NTDT |
| LS Z-score–Women | -0.20 ± 0.96 | -1.99 ± 1.00 | -1.89 ± 1.04 | <0.001 | Control vs TDT |
|  |  |  |  | <0.001 | Control vs NTDT |
|  |  |  |  | 0.473 | TDT vs NTDT |
| FN Z-score–All | 0.45 ± 0.87 | -1.15 ± 0.75 | -1.13 ± 0.92 | <0.001 | Control vs TDT |
|  |  |  |  | <0.001 | Control vs NTDT |
|  |  |  |  | 0.639 | TDT vs NTDT |
| FN Z-score–Men | 0.65 ± 0.88 | -0.97 ± 0.85 | -0.78 ± 0.65 | <0.001 | Control vs TDT |
|  |  |  |  | <0.001 | Control vs NTDT |
|  |  |  |  | 0.729 | TDT vs NTDT |
| FN Z-score–Women | 0.31 ± 0.85 | -1.24 ± 0.69 | -1.46 ± 1.04 | <0.001 | Control vs TDT |
|  |  |  |  | <0.001 | Control vs NTDT |
|  |  |  |  | 0.488 | TDT vs NTDT |

Abbreviations: Trabecular Bone Score: TBS; Lumbar Spine Z-score: LS Z-score; Femoral Neck Z-score: FN Z-score; Transfusion-Dependent Thalassemia: TDT; Non–Transfusion-Dependent Thalassemia: NTDT

**Supplementary Table 2. Multiple regression analysis of factors influencing trabecular bone score**

| Variable | Model 1 | | | Model 2 | | | Model 3 | | | Model 4 | | Model 5 | |
| --- | --- | --- | --- | --- | --- | --- | --- | --- | --- | --- | --- | --- | --- |
|  | β | 95%CI | β | | 95%CI | β | | 95%CI | β | | 95%CI | β | 95%CI |
| Intercept | 1.401^*^ | [1.304, 1.499] | 1.403^*^ | | [1.305, 1.501] | 1.464^*^ | | [1.383, 1.545] | 1.443^*^ | | [1.353, 1.533] | 1.478* | [1.397, 1.559] |
| Age  (per 1 year increase) | -0.002^*^ | [-0.003, -0.001] | -0.002^*^ | | [-0.003, -0.001] | -0.003^*^ | | [-0.004, -0.002] | -0.003^*^ | | [-0.004, -0.002] | -0.003* | [-0.004, -0.002] |
| Men | Ref | Ref | Ref | | Ref | Ref | | Ref | Ref | | Ref | Ref | Ref |
| Women | -0.015 | [-0.043, 0.013] | -0.014 | | [-0.042, 0.014] | -0.012 | | [-0.035, 0.011] | 0.003 | | [-0.023, 0.029] | -0.007 | [-0.030, 0.016] |
| BMI  (per 1 kg/m^2^ increase) | 0.004 | [-0.000, 0.009] | 0.004 | | [-0.000, 0.009] | 0.003 | | [-0.000, 0.007] | 0.002 | | [-0.002, 0.006] | 0.003 | [-0.001, 0.006] |
| Control group | Ref | Ref | Ref | | Ref | Ref | | Ref | Ref | | Ref | Ref | Ref |
| Thalassemia | -0.143^*^ | [-0.174, -0.111] | - | | - | - | | - | - | | - | - | - |
| NTDT | - | - | -0.127^*^ | | [-0.173, -0.082] | -0.039 | | [-0.081, 0.003] | -0.052^*^ | | [-0.101, -0.003] | -0.013 | [-0.058, 0.031] |
| TDT | - | - | -0.151^*^ | | [-0.186, -0.115] | -0.047^*^ | | [-0.083, -0.012] | -0.080^*^ | | [-0.120, -0.041] | -0.033 | [-0.071, 0.004] |
| LS Z-score  (per 1 increase) | - | - | - | | - | 0.061^*^ | | [0.049, 0.073] | - | | - | 0.019^*^ | [0.004, 0.034] |
| FN Z-score (per 1 increase) | - | - | - | | - | - | | - | 0.044^*^ | | [0.029, 0.059] | 0.053^*^ | [0.040, 0.067] |

Model descriptions: Model 1: age, sex, BMI and thalassemia vs control; Model 2: age, sex, BMI, TDT vs control and NTDT vs control; Model 3: age, sex, BMI, TDT vs control, NTDT vs control and LS Z-score; Model 4: age, sex, BMI, TDT vs control, NTDT vs control and FN Z-score; Model 5: age, sex, BMI, TDT vs control, NTDT vs control, LS Z-score and FN Z-score.

*Statistically significant at p < 0.05.

Abbreviations: TBS: trabecular bone score; BMI: body mass index; LS Z-score: lumbar spine Z-score; FN Z-score: femoral neck Z-score; TDT: transfusion-dependent thalassemia; NTDT: non–transfusion-dependent thalassemia; Ref: reference group.

**Supplementary Table 3.** **Characteristics of thalassemia patients stratified by transfusion status and splenectomy**

| **Variable** | **TDT/splenectomy** (N = 34) | **NTDT/splenectomy (N = 4)** | **TDT/no splenectomy (N = 22)** | **NTDT/no splenectomy (N = 25)** |
| --- | --- | --- | --- | --- |
| Age (years) | 29.59 ± 8.84 | 32.50 ± 11.15 | 32.05 ± 12.70 | 36.00 ± 14.60 |
| Female sex | 21 (61.8%) | 2 (50.0%) | 16 (72.7%) | 12 (48.0%) |
| BMI (kg/m^2^) | 18.73 ± 2.12 | 18.52 ± 3.76 | 19.40 ± 2.25 | 19.69 ± 1.98 |
| Ferritin | 2860.76 ± 2744.57^b^ | 2881.00 ± 3342.81 | 2524.77 ± 1994.38^c^ | 1454.20 ± 1194.59^b, c^ |
| Fracture history | 16 (47.1%)^b^ | 2 (50.0%) | 7 (31.8%) | 5 (20.0%)^b^ |
| TBS | 1.240 ± 0.120^b^ | 1.195 ± 0.125 | 1.295 ± 0.113 | 1.292 ± 0.139^b^ |
| LS Z-score | -2.22 ± 1.02^a,b^ | -2.55 ± 1.17 | -1.45 ± 0.79^a^ | -1.62 ± 0.88^b^ |
| FN Z-score | -1.31 ± 0.78 | -1.67 ± 1.82 | -0.97 ± 0.66 | -1.07 ± 0.80 |

^a^Statistically significant difference (p < 0.05): TDT/splenectomy vs. TDT/no splenectomy
^b^Statistically significant difference (p < 0.05): TDT/splenectomy vs. NTDT/no splenectomy groups
^c^Statistically significant difference (p < 0.05): TDT/ no splenectomy vs. NTDT/no splenectomy groups

Abbreviations: TDT: transfusion-dependent thalassemia; NTDT: non–transfusion-dependent thalassemia; BMI: body mass index; TBS: trabecular bone score; LS: lumbar spine; FN: femoral neck
